# How to promote student creativity through AI in higher education: the role of students’ attitude and digital competencies

**DOI:** 10.3389/fpubh.2026.1772946

**Published:** 2026-02-11

**Authors:** Feifei Hao, Zhanyou Wang, Liang Ma, Hui Zhao, Xinkui Liu, Xin Zhao, Na Zhang, Xia Li

**Affiliations:** 1 College of Traditional Chinese Medicine, Shandong University of Traditional Chinese Medicine, Jinan, China; 2 School of Labor Relations, Shandong Management University, Jinan, China; 3 School of Management Science and Engineering, Shandong University of Finance and Economics, Jinan, China; 4 School of Health, Shandong University of Traditional Chinese Medicine, Jinan, China; 5 Innovation Research Institute of Traditional Chinese Medicine, Shandong University of Traditional Chinese Medicine, Jinan, China; 6 School of Pharmacy, Shandong University of Traditional Chinese Medicine, Jinan, China; 7 College of Acupuncture and Moxibustion and Tuina, Shandong University of Traditional Chinese Medicine, Jinan, China

**Keywords:** AI, AI used for entertainment, AI used for learning, attitudes toward AI, digital competencies, student creativity

## Abstract

Artificial intelligence (AI) is increasingly integrated into higher education, yet how different purposes of AI use influence student creativity remains underexplored. In particular, little is known about the mediating role of digital competencies and the moderating role of students’ attitudes toward AI. Drawing on Social Cognitive Theory, this study examines how AI use for learning and AI use for entertainment relate to student creativity through digital competencies, and how attitudes toward AI condition these relationships. Data were collected from 271 undergraduate students majoring in Traditional Chinese Medicine in China and analyzed using PLS-SEM and moderated mediation analysis. The results show that both learning-oriented and entertainment-oriented AI use positively relate to digital competencies, which in turn enhance student creativity. Digital competencies fully mediate the relationship between AI use for learning and creativity and partially mediate the relationship between AI use for entertainment and creativity. Moreover, attitudes toward AI play a dual moderating role: positive attitudes strengthen the effect of entertainment-oriented AI use but weaken the effect of learning-oriented AI use on digital competencies. This study contributes to the literature by distinguishing different purposes of AI use, identifying digital competencies as a key explanatory mechanism, and revealing the nuanced role of attitudes toward AI in shaping creativity outcomes. It also offers practical implications for designing AI-supported educational practices in specialized domains such as Traditional Chinese Medicine.

## 1 Introduction

In the current digital era, the application of artificial intelligence (AI) in the field of education is becoming increasingly widespread (1), demonstrating great potential (2). AI driven tools, such as intelligent tutoring systems and educational Chatbots, are gradually transforming the way of teaching and learning by providing students with immediate access to information, personalized feedback, and diverse problem-solving resources (3). Generative AI has introduced new possibilities for personalized and creative learning experiences in Traditional Chinese Medicine (TCM). Incorporating these technological developments into the analysis not only enhances the theoretical depth of the study but also strengthens its practical implications for modernizing TCM education. However, the application of AI in education also poses many challenges to the development of students’ creativity (4). On one hand, the convenient information access provided by AI may lead to students’ over reliance, thus reducing their opportunities for active thinking and exploration. On the other hand, the accuracy and reliability of AI generated content also have certain problems, which may mislead students and hinder the cultivation of their creative thinking.

Current research on students’ creativity mainly focuses on aspects such as the methods of creativity cultivation, influencing factors, and the relationship with academic performance (5, 6). In terms of cultivation methods, research has explored the promoting effects of project based learning, heuristic teaching, on students’ creativity (7). Regarding influencing factors, it pays attention to the impacts of students’ personality traits, family environment, school atmosphere, and other factors on creativity (8, 9). Despite the growing interest in AI and creativity, current research presents several important limitations. First, most studies treat AI use as a homogeneous construct and rarely distinguish between different purposes of use. In practice, students employ AI for both learning-oriented purposes (e.g., solving academic problems, acquiring knowledge, and supporting coursework) and entertainment-oriented purposes (e.g., relaxation, gaming, and enjoyment). These two types of use involve fundamentally different cognitive processes and motivational orientations, which may lead to different consequences for creativity. Second, existing studies have paid limited attention to the mediating mechanisms that explain how AI use translates into creativity. In particular, students’ digital competencies, their ability to effectively use, evaluate, and integrate digital technologies (10), have been largely overlooked as a potential explanatory pathway. Third, prior research seldom considers the contingent role of students’ attitudes toward AI. Attitudes reflect individuals’ cognitive and emotional evaluations of AI and shape how actively and effectively they engage with AI tools (11, 12). Ignoring this factor limits our understanding of when and for whom AI use is most beneficial for developing creativity.

Drawing on Social Cognitive Theory (SCT), this study provides a theoretically grounded framework to address these gaps. SCT emphasizes the reciprocal interaction among environmental factors, personal cognition, and behavioral outcomes. In the context of this study, AI use represents an environmental stimulus, digital competencies capture students’ cognitive capabilities in interacting with digital technologies, attitudes toward AI reflect personal evaluative beliefs, and creativity constitutes a key behavioral outcome. This framework enables us to explain not only whether AI use is associated with student creativity, but also how and under what conditions such associations emerge. Accordingly, this study investigates how different purposes of AI use (AI used for learning and AI used for entertainment) influence student creativity through the mediating role of digital competencies and how these relationships are moderated by students’ attitudes toward AI. This research offers three main contributions. First, it differentiates AI use by purpose and demonstrates that learning-oriented and entertainment-oriented AI use follow distinct pathways in shaping creativity. Second, it identifies digital competencies as a critical mediating mechanism linking AI use and student creativity. Third, it reveals the dual moderating role of attitudes toward AI, showing that positive attitudes do not uniformly strengthen AI effects but operate differently across learning and entertainment contexts. By doing so, this study advances theoretical understanding of AI-enabled creativity and provides practical guidance for designing more effective AI-supported educational practices in specialized domains such as TCM education.

## 2 Theoretical background and hypothesis development

### 2.1 AI use practice and purposes

AI is being increasingly used in the education sector (Table 1), and its applications can be mainly categorized into two types: learning and entertainment. The impact of these two types of applications on students’ creativity has drawn significant attention, and existing research has conducted numerous explorations (2). AI used for learning refers to the application of AI technology to assist students in acquiring knowledge, enhancing skills, promoting understanding, and reinforcing learning content (13). It encompasses various aspects such as intelligent tutoring, providing learning resources, and facilitating teaching, aiming to optimize the learning process and improve learning outcomes. Existing research in this area mainly focuses on intelligent tutoring, learning resource provision, and teaching assistance (14, 15). Regarding the impact on students’ creativity, on one hand, the abundant resources provided by AI can broaden students’ knowledge horizons and stimulate innovative thinking (16). On the other hand, excessive reliance on AI may lead to a decline in students’ independent thinking ability and thus inhibit the development of creativity (17). AI used for entertainment refers to the use of AI technology to create entertaining experiences for students, integrating educational elements into the entertainment process to achieve learning through entertainment. Existing research in this area mainly focuses on gamified learning and virtual experiences (18, 19). As for the impact on students’ creativity, on the positive side, it can stimulate students’ curiosity and exploration desire, allowing them to learn and create in an interesting environment (2). On the negative side, if the entertainment component is overly emphasized, it may distract students from learning and prevent them from focusing on knowledge acquisition and creativity development (16).

**Table 1:** AI’s practice in reshaping creativity across different disciplines.

Discipline	AI Tools	Manifestations of creativity enhancement	Practice
Humanities and Social Sciences	GPT-4 tool	Identifying implicit social patterns from massive texts to innovate theoretical models	Sociology students analyzed 5,000 Ming and Qing dynasty county records using AI, uncovering the correlation mechanism of climate change-population migration-folk beliefs, and published a paper.
Art and Design	Procreate AI brushes	Breaking through medium limitations to create immersive interactive art	Digital media students used AI to generate dynamic murals that change with audience emotions, installed in urban public spaces, triggering hot discussions on social media.
Traditional Chinese Medicine (TCM)	TCM AI-assisted diagnosis systems, VR anatomy tools	Innovating diagnostic protocols and promoting modernization of traditional medicine	Students from Beijing University of Chinese Medicine proposed an AI-assisted syndrome differentiation-precision moxibustion scheme based on AI analysis of 100,000 clinical cases, which was approved as a national college student innovation project.

Recent studies have increasingly emphasized the role of frontier technologies such as generative AI, virtual reality (VR), and blockchain in education and creativity research. Generative AI has been shown to facilitate personalized learning and foster creative thinking by providing adaptive feedback and content generation (20). VR has been applied to create immersive learning environments that enhance experiential training, while blockchain has been explored for its potential to ensure secure data management and transparent credentialing in higher education (21, 22). However, despite these promising developments, very limited research has systematically examined the application of these technologies within the context of Traditional Chinese Medicine (TCM) education. This gap highlights the originality and necessity of the present study, which aims to explore how AI-enabled approaches can contribute to creativity development in specialized domains such as TCM.

### 2.2 Social cognitive theory

Social Cognitive Theory (SCT) emphasizes the dynamic interplay between individual behavior, environmental influences, and personal factors (23). It posits that learning occurs through observation, imitation, and modeling, with individuals developing capabilities by observing others’ behaviors and outcomes (24). SCT highlights the importance of self-efficacy, the belief in one’s ability to succeed in specific tasks, which influences motivation and behavior (25). In the context of this study, SCT provides a robust framework to understand how AI use associated with student creativity through digital competencies. AI tools, when used for learning or entertainment, create an environment where students observe and model behaviors related to digital engagement (26, 27). This exposure enhances their digital competencies, which in turn fosters creativity. Additionally, students’ attitudes toward AI reflect their self-efficacy beliefs, influencing how effectively they engage with AI tools. Thus, SCT explains the observed relationships between AI use, digital competencies, and creativity by emphasizing the role of environmental influences (AI tools), personal factors (attitudes), and behavioral outcomes (creativity). Beyond serving as a general explanatory framework, SCT in this study is extended to conceptualize how different types of AI use function as distinct environmental stimuli, how digital competencies operate as a core cognitive capability, and how attitudes toward AI shape the effectiveness of these stimuli. In doing so, this study advances SCT by specifying a technology-centered mechanism through which environmental factors are transformed into creative outcomes in AI-supported educational contexts.

### 2.3 AI use and digital competencies

Student digital competencies refer to the set of skills, knowledge, and attitudes that enable students to effectively use digital technologies for learning, communication, problem solving, and creative expression in various contexts (28). Traditional tools emphasize operational proficiency (e.g., mastering microscope adjustments), while AI tools prioritize digital literacy in the intelligent era, such as understanding how AI processes data, evaluating model accuracy, and optimizing algorithm parameters (Table 2). Thus, investigate the relationship between AI and digital competencies is particularly important.

**Table 2:** Digital capability from function substitution to intelligent empowerment.

Aspect	Traditional educational tools	AI Tools
Core technology	Mechanical/electrical technology (e.g., projectors, lab equipment) or static digital tools (e.g., PDFs, basic software).	Machine learning, big data analytics, natural language processing (NLP), and computer vision.
Role in digital competency	Primarily assist in knowledge presentation or simple data processing, with limited requirement for digital skills.	Require users to master algorithmic thinking, data interpretation, and human-computer collaboration (e.g., training AI models, debugging neural networks).

When student use AI for learning purposes, it may positively associate with student digital competencies. The reasons maybe that: First, AI tools can provide students with personalized learning pathways and resources, helping them acquire knowledge and skills more efficiently (29, 30). For example, AI driven learning platforms can tailor content based on individual progress and abilities, enhancing students’ proficiency in using digital tools and learning outcomes. Additionally, AI’s intelligent feedback mechanisms help student correct mistakes in real time, strengthening their self-directed learning and problem solving abilities (31), which are key components of digital competencies. Moreover, AI can create enriched and interactive learning environments through virtual labs and online collaboration tools, further boosting students’ digital literacy. These factors collectively make AI a powerful driver for enhancing student digital competencies in learning contexts. Based on above discussions, this paper proposed the following hypothesis:

*H1*: AI used for learning has a positive effect on student digital competencies.

Another important purpose student use AI is for entertainment purposes (32). The logic behind AI used for entertainment positively associates with student digital competencies is mainly realized through several variables. First, AI driven entertainment content (such as intelligent games and virtual reality experiences) typically requires students to possess certain digital operation skills, such as using complex software or hardware (33). This interactivity prompts students to continuously learn and adapt to new digital tools and technologies while being entertained, thereby improving their digital competencies. Second, creative elements in AI entertainment applications (such as programming games or digital art creation tools) can stimulate students’ creativity and digital literacy (34). Through these tools, students not only enjoy entertainment but also learn and apply digital skills in practice. Furthermore, AI in entertainment can help students better understand and utilize digital resources through recommendation systems and personalized experiences (35), further enhancing their digital competencies. This subtle influence in entertainment makes AI an important pathway for improving student digital competencies. Based on above discussions, this paper proposed the following hypothesis:

*H2*: AI used for entertainment has a positive effect on student digital competencies.

### 2.4 Digital competencies and student creativity

Student creativity refers to the ability of students to generate novel, original, and valuable ideas or solutions in various contexts (36). It involves the capacity to think divergently, explore multiple possibilities, and apply imaginative thinking to solve problems or create new products. Digital competencies may positively influence student creativity through enhanced information processing and collaborative abilities. First, digital competencies enable students to access, filter, and integrate information more efficiently, providing a rich pool of resources for creative thinking (10). For example, students can leverage online resources to gather cross disciplinary knowledge, which can spark new ideas and solutions. Second, digital tools support collaborative learning, allowing students to interact with diverse peers in virtual teams. This multicultural exchange fosters creative collisions and innovation. Additionally, digital competencies facilitate the expression and presentation of creative ideas, such as using multimedia tools to visualize abstract concepts (37), thereby enhancing the realization and dissemination of creativity. Therefore, digital competencies positively associate with student creativity through variables like information integration, collaborative interaction, and creative expression. Based on above discussions, this paper proposed the following hypothesis:

*H3*: Digital competencies has a positive effect on student creativity.

### 2.5 Moderating role of attitude toward AI

Attitude toward AI is defined as an individual’s cognitive, emotional, and behavioral tendency toward artificial intelligence (12). This paper posits that the more positive students’ attitudes toward artificial intelligence (AI), the weaker the effect of AI on enhancing students’ digital competencies during the learning process. This negative moderating effect may be based on the following points: When students hold a highly positive attitude toward AI, they may become overly reliant on the instant feedback and solutions provided by AI (38), thereby reducing opportunities for independent thinking and problem solving (39, 40). This dependency may lead to a lack of independent analysis and problem solving skills when students face complex issues, thus weakening the enhancement of their digital competencies. For example, students may habitually rely on AI’s suggestions rather than actively exploring and learning new skills and knowledge. A positive attitude may also shift students’ learning motivation more toward the process of using AI rather than the learning content itself (41, 42). This shift in motivation may cause students to focus more on how to use AI during the learning process, rather than on how to enhance their digital competencies through AI. For example, students may enjoy the process of interacting with AI more than deeply understanding and applying the knowledge and skills provided by AI. Based on above discussions, this paper proposed the following hypothesis:

*H4*: Attitude toward AI acts as a negative moderating role between AI used for learning and student digital competencies.

This paper proposes that students’ attitudes toward artificial intelligence (AI) play a positive moderating role between AI used for entertainment and student digital competencies. This means that when students hold positive attitudes toward AI, its application in entertainment contexts may more effectively promote the development of students’ digital competencies. The rationale behind this logic is that a positive attitude can enhance students’ acceptance and willingness to use AI tools (41), especially in entertainment contexts where AI applications are often more engaging and enjoyable. For example, through AI driven games or interactive entertainment applications, students may unconsciously improve their digital skills (43, 44), such as data analysis, problem solving, and logical thinking, while enjoying entertainment. Moreover, a positive attitude may also encourage students to actively explore more functionalities of AI (33), thereby further enhancing their digital literacy. Therefore, students’ positive attitudes toward AI may amplify the association between AI and digital competencies in entertainment contexts, making it an effective learning tool. Based on above discussions, this paper proposed the following hypothesis:

*H5*: Attitude toward AI acts as a positive moderating role between AI used for entertainment and student digital competencies.

**Figure 1:**
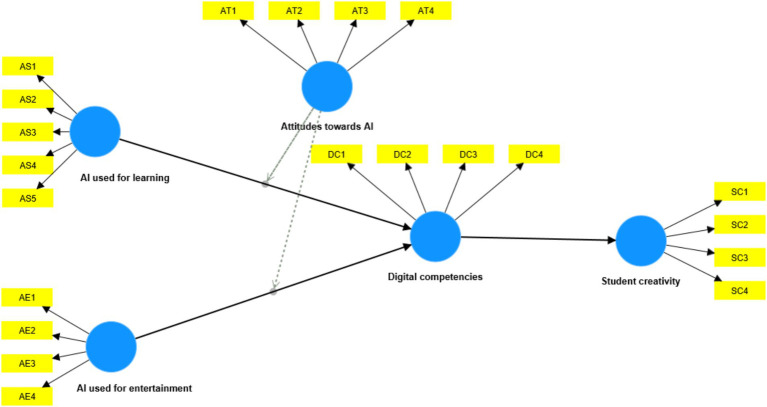
Research model.

Based on the above discussion, the proposed research model is presented in Figure 1.

## 3 Research methods

### 3.1 Measures

In this paper, a questionnaire was used to collect data. Each measurement item was drawn from previous literature (see Table 3). AI used for learning (AL) included five items and was taken from (45). AI used for entertainment (AE) included four items and was taken from Ali-Hassan, Nevo and Wade (46). Attitudes toward AI (AT) was measured with four items derived from Shao, Nah, Makady and McNealy (47). Digital competencies (DC) was measured with four items derived from David, Zinica, Barbuta-Misu, Savga and Virlanuta (48). Student creativity (SC) included four items and was taken from Jia, Luo, Fang and Liao (49). All of the above items were scored on a seven-point Likert scale ranging from 1 (strongly disagree/unlikely) to 7 (strongly agree/likely). In this study, all measurement scales were either adopted or adapted from well-established instruments in prior literature. For scales originally developed in non-educational or organizational contexts (e.g., AI used for entertainment), an adaptation process was conducted to ensure their suitability for the TCM undergraduate learning environment. Specifically, all items were carefully reworded to fit the educational context by replacing work-related expressions (e.g., “job” or “work”) with study-related expressions (e.g., “learning” or “study”). To enhance content validity, the adapted questionnaire was reviewed by two scholars specializing in educational technology and one expert in Traditional Chinese Medicine (TCM) education, who evaluated the clarity, relevance, and contextual appropriateness of each item. In addition, a pilot test was conducted with 30 TCM undergraduate students to examine item comprehension and wording suitability. Minor revisions were made based on their feedback. Furthermore, the validity of all adapted scales was empirically supported by the results of the measurement model. All constructs demonstrated high factor loadings, strong internal consistency reliability, and satisfactory convergent and discriminant validity, indicating that the adapted instruments were appropriate for the TCM educational context. This adaptation approach is consistent with prior studies that have successfully transferred technology-use and digital behavior scales from organizational settings to educational contexts (50), supporting the feasibility of contextual scale adaptation.

**Table 3:** Measurement Items.

Constructs	Items measured
AI used for learning (45)	AL1. I use AI tool to obtain ideas and participate in TCM learning-related discussion.
	AL2. I use AI tool to acquire solutions for TCM learning problems.
	AL3. I use AI tool in my daily learning to ask TCM questions.
	AL4. I use AI tool in my daily learning to get TCM knowledge.
	AL5. I use AI tool in my daily learning to assist my TCM learning.
AI used for entertainment Ali-Hassan et al.(46)	AE1. I use AI tool in my daily learning to enjoy my break
	AE2. I use AI tool in my daily learning to take a break from work
	AE3. I use AI tool in my daily learning to entertain myself
	AE4. I use AI tool in my daily learning to relax at work
Attitudes toward AI Shao et al.(47)	AT1. I think the use of AI technology is good.
	AT2. I think the use of AI technology is wise.
	AT3. I think the use of AI technology is favorable.
	AT4. I think the use of AI technology is beneficial.
Digital competencies David et al.(48)	DC1. I take advantage of any opportunity to attend a training program focused on the development of digital competencies.
	DC2. I conduct research on how AI-based platforms and Web 3 technologies (block chain, smart contracts, and decentralized organization) will affect the responsibilities of managers and the tasks of public administration employees.
	DC3. I respond positively when asked to implement digital technologies in internal processes or in communication with external partners (citizens, companies, and institutions).
	DC4. As often as possible, I use the digital resources or technologies that are available to me because I believe that these digital resources make the processes or tasks that I have to perform easier.
Student creativity Jia et al.(49)	SC1. I seek new ideas and ways to solve problems.
	SC2. I try new ideas or methods first.
	SC3. I generate ground-breaking ideas related to the field.
	SC4. I generate new inventions and applications.

### 3.2 Data collection

Considering that generative artificial intelligence is currently the most widely used type of AI among college students in TCM schools, the AI investigated in this paper primarily refers to generative AI. This study conducted data collection through an online questionnaire to efficiently obtain rich data. The samples were sourced from undergraduate students majoring in TCM at a university of shandong traditional Chinese medicine. TCM programs in China share similar curricula. After designing the questionnaire on the platform, we asked the counselors to invite undergraduate students from the college to complete it. The questionnaire was distributed through Soujump, an online survey platform, which facilitated easy access and completion by the respondents. This platform is widely used by previous scholars (51). In addition, the platform only allowed one questionnaire to be completed by each IP address, which prevented duplicate questionnaires from being completed by the same respondent. After the twoweek data collection process, this study collected 271 completed questionnaires. The final valid sample consisted of 271 undergraduate students majoring in Traditional Chinese Medicine. To further evaluate whether this sample size was sufficient for the complexity of the moderated-mediation model, a *post hoc* power analysis was conducted using G*Power 3.1. With N = 271, *α* = 0.05, and five predictors, the achieved power to detect a medium effect size (f^2^ = 0.15) was approximately 1.00. This indicates that the sample size was more than adequate to detect medium effects, although very small effects may not be reliably captured.

The descriptive statistics of respondent characteristics are presented as follows (Table 4). Based on the demographic data from questionnaire, the gender distribution revealed that males comprised 32.1% of the respondents, while females accounted for 67.9%. Regarding their educational backgrounds, freshmen made up 33.58% of the sample, followed by a minimal 0.74% representation from sophomores. Juniors constituted the largest group at 64.21%, with both seniors and those pursuing master’s degrees or higher each contributing another 0.74%. When examining the participants’ experience with artificial intelligence tools, it was found that 33.58% had less than 1 year of experience, 32.84% had between one and 2 years, 15.87% had between 3 and 5 years, and 14.76% had more than 5 years of experience. In terms of the purposes for using artificial intelligence, a significant 77.12% of respondents utilized AI for learning, while 20.66% used it for entertainment, and a smaller portion of 2.21% cited other purposes.

**Table 4:** Descriptive statistics of respondents.

Characteristics	Category	Frequency	Percentage (%)
Gender	Male	87	32.1
	Female	184	67.9
Educational background	Freshman	91	33.58
	Sophomore	2	0.74
	Junior	174	64.21
	Senior	2	0.74
	Master’s degree or above	2	0.74
Experience with AI tools	Less than 1 year	91	33.58
	1–2 years	89	32.84
	3–5 years	43	15.87
	More than 5 years	40	14.76
Purpose of AI use	Learning	209	77.12
	Entertainment	56	20.66
	Other	6	2.21

## 4 Data analysis and results

This study employed SmartPLS 4.0 for the analysis of both measurement and structural models. In contrast to CB-SEM, PLS-SEM requires a smaller sample size and does not demand normal distribution of the data, making it a more suitable choice for the exploratory analyses and formative constructs explored in this research. The study adopted a twophase approach: initially assessing the measurement model, and subsequently examining the relationships among the latent variables within the structural model.

### 4.1 Measurement model

This study assessed the reliability and validity of the measurement model. Specifically, as demonstrated in Table 5, Cronbach’s alpha coefficients for the constructs fell within a range of 0.887 to 0.924, and composite reliability values ranged from 0.922 to 0.946, both exceeding the 0.7 threshold, thus indicating solid reliability. In terms of validity, the study evaluated both convergent and discriminant validity. The average variance extracted (AVE) for all constructs exceeded 0.7, and all factor loadings listed in Table 6 surpassed the 0.7 threshold, indicating strong convergent validity. Additionally, the square root of each construct’s AVE, as shown in Table 5, exceeded its correlations with other constructs.

**Table 5:** Descriptive statistics and inter-construct correlations.

Items	Cronbach’s alpha	CR (rho_a)	CR (rho_c)	AVE	AE	AL	AT	DC	SC
AE	0.921	0.927	0.944	0.809	**0.899**				
AL	0.924	0.926	0.943	0.767	0.507	**0.876**			
AT	0.924	0.926	0.946	0.814	0.479	0.617	**0.902**		
DC	0.887	0.890	0.922	0.747	0.479	0.502	0.741	**0.864**	
SC	0.888	0.889	0.923	0.750	0.482	0.443	0.696	0.843	**0.866**

AI used for learning (AL), AI used for entertainment (AE), Attitudes toward AI (AT), Digital competences (DC), Student creativity (SC). The diagonal (bold) elements are the square roots of AVEs, and off-diagonal elements are correlations between constructs.

**Table 6:** Cross loadings.

Items	AE	AL	AT	DC	SC
AE1	**0.880**	0.535	0.409	0.374	0.407
AE2	**0.920**	0.490	0.417	0.436	0.417
AE3	**0.904**	0.421	0.417	0.424	0.419
AE4	**0.893**	0.394	0.472	0.479	0.481
AL1	0.379	**0.851**	0.496	0.400	0.325
AL2	0.413	**0.904**	0.578	0.459	0.411
AL3	0.469	**0.851**	0.495	0.445	0.408
AL4	0.445	**0.884**	0.547	0.438	0.373
AL5	0.511	**0.887**	0.580	0.455	0.418
AT1	0.383	0.534	**0.879**	0.643	0.609
AT2	0.474	0.570	**0.914**	0.658	0.622
AT3	0.438	0.559	**0.903**	0.639	0.605
AT4	0.433	0.564	**0.911**	0.725	0.671
DC1	0.408	0.400	0.660	**0.851**	0.709
DC2	0.450	0.345	0.538	**0.852**	0.685
DC3	0.393	0.505	0.661	**0.893**	0.726
DC4	0.410	0.475	0.689	**0.860**	0.787
SC1	0.329	0.387	0.617	0.700	**0.815**
SC2	0.389	0.413	0.639	0.684	**0.883**
SC3	0.427	0.356	0.570	0.776	**0.895**
SC4	0.520	0.380	0.587	0.758	**0.870**

The bold font represents factor loadings.

### 4.2 Structural model

**Figure 2:**
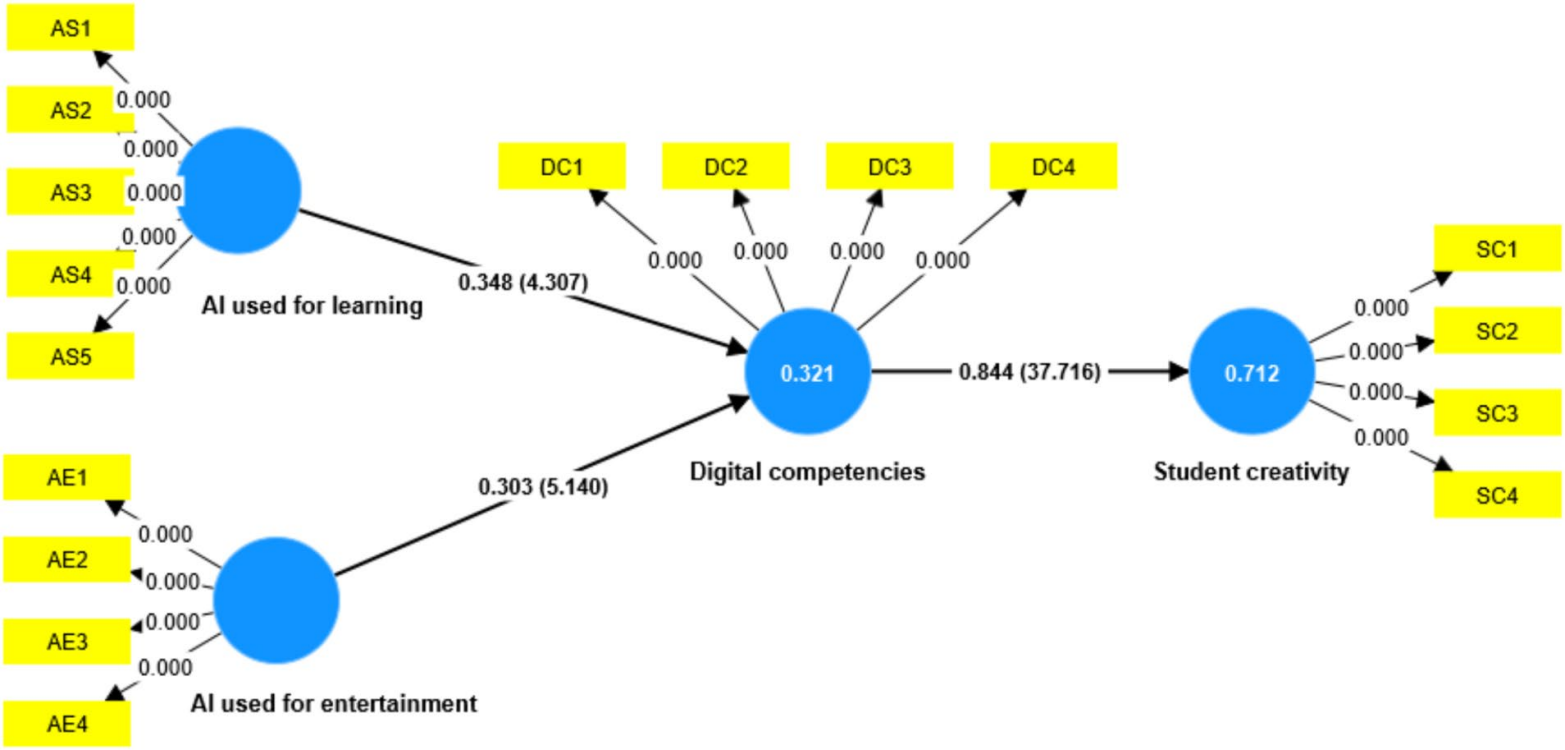
Results of the structural model analysis.

To evaluate the overall model fit, multiple fit indices were examined, including SRMR, NFI, d_ULS, and d_G. As shown in Table 7, the SRMR value of 0.060 is below the recommended threshold of 0.08, indicating a good approximate model fit. The NFI value of 0.838 exceeds the acceptable cut-off of 0.70, suggesting an adequate incremental fit. Moreover, both d_ULS and d_G are lower than their corresponding 95% bootstrap quantiles, further supporting the satisfactory fit of the proposed model. Taken together, these results demonstrate that the overall model fit is acceptable and suitable for hypothesis testing as shown in Figure 2. Specifically, the results showed that AI used for learning positively associated with digital competences (*β* = 0.348, *T* = 4.307), indicating H1 is supported. AI used for entertainment positively associated with digital competences (*β* = 0.303, *T* = 5.140), indicating H2 is supported. Digital competence (*β* = 0.844, *T* = 37.716) positively associated with student creativity, thus supporting H3.

**Table 7:** Model of Fit.

Items	Saturated model	Estimated model
SRMR	0.056	0.060
d_ULS	0.722	0.841
d_G	0.556	0.560
Chi-square	881.777	858.574
NFI	0.833	0.838

### 4.3 Mediating effects

To test the mediating effect, hypothesis testing was carried out using PROCESS in SPSS with the bias-corrected method and the percentile method at a 95% confidence interval (52). In this approach, the mediating effect of the mediator variable was supported when the confidence interval for the indirect effect does not include zero. A distinction was also made between full and partial mediation depending on whether the confidence interval for the direct effect includes zero. As Table 8 shown, digital competences completely mediated the relationship between AI used for learning and student creativity. Besides, digital competences partially mediated the relationship between AI used for entertainment and student creativity. These results indicated that AI used for learning and AI used for entertainment directly affect student creativity and indirectly through digital competences.

**Table 8:** Mediating effects results.

M/(IV)/(DV)	Items	Effect	Coefficient		Bias-Corrected		Percentile		Mediation existence
			SE	T	95%CI		95%CI		
DC/(AL)/(SC)	Direct effect	0.031	0.038	0.802	−0.045	0.106	−0.045	0.106	Complete
	Indirect effect	0.411	0.067	6.134	0.284	0.540	0.285	0.542	
DC/(AE)/(SC)	Direct effect	0.101	0.037	2.709	0.028	0.174	0.028	0.174	Partial
	Indirect effect	0.377	0.052	7.250	0.282	0.491	0.276	0.485	

### 4.4 Moderating effects

**Figure 3:**
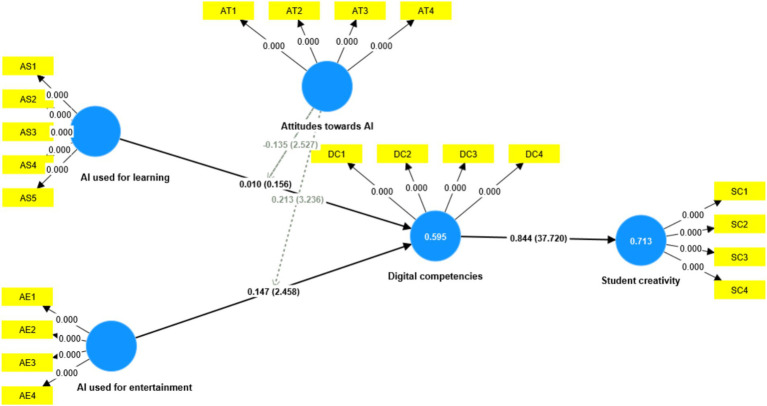
Results of the moderating effects.

**Figure 4:**
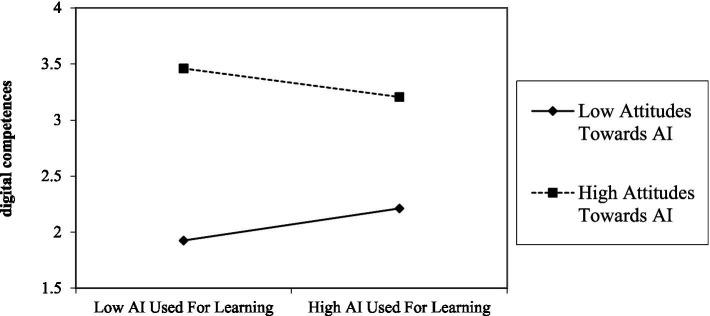
Moderating role of attitudes toward AI between AI used for learning and digital competences.

**Figure 5:**
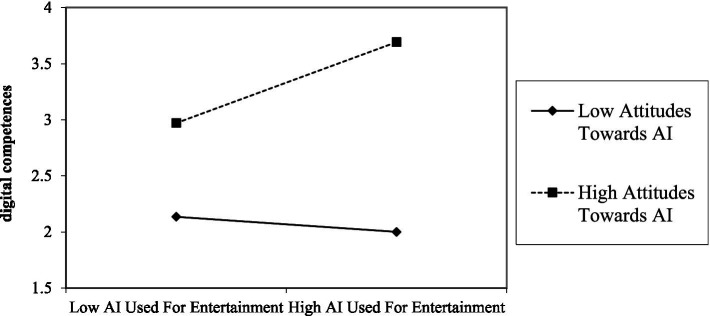
Moderating role of attitudes toward AI between AI used for entertainment and digital competences.

As shown in Table 9, this study conducted multilevel regression analyses of the moderating effects of attitudes toward AI. The results showed that attitudes toward AI negatively moderated the relationship between AI used for learning and digital competences (*β* = −0.135, *T* = 2.527) as Figure 3 showing, implying that H9a was supported. This moderating effect is further illustrated in Figure 4. In addition, the results showed that attitudes toward AI positively moderated the relationship between AI used for entertainment and digital competences (*β* = 0.213, *T* = 3.236) as Figure 3 showing, implying that H9b was supported. This moderating effect is further illustrated in Figure 5.

**Table 9:** Moderating effect of attitudes toward AI.

No	Hypothesized relationship	Path coefficient	t-statistics	*p*-values	Lower CI	Upper CI	Decision
H9a	AL*AT → DC	−0.135	2.527	0.011	−0.237	−0.029	Supported
H9b	AE*AT → DC	0.213	3.236	0.001	0.091	0.346	Supported

### 4.5 Importance–performance map analysis (IPMA) results

**Figure 6:**
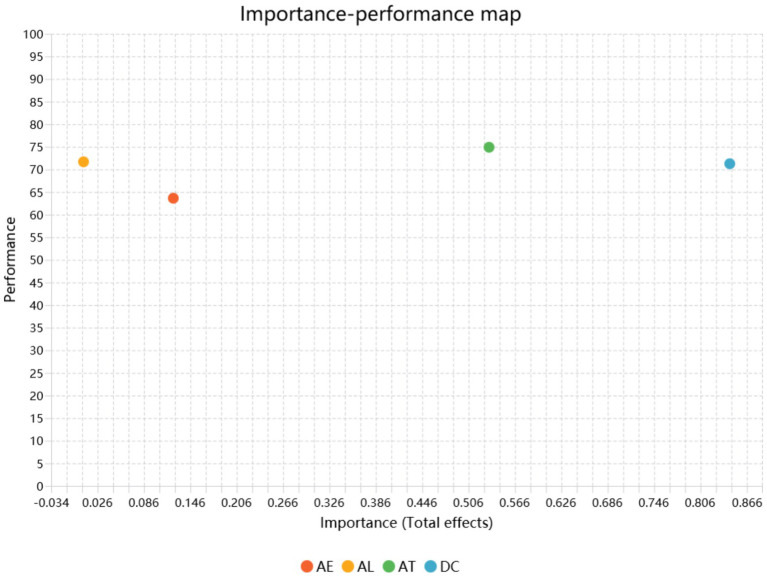
Importance–performance map analysis (IPMA) results.

The IPMA results (see Figure 6) provide nuanced insights into how the four predictors, AI used for learning (AL), AI used for entertainment (AE), attitudes toward AI (AT), and digital competencies (DC), contribute to student creativity (SC) in terms of both importance (total effects) and performance.

Overall, AT and DC emerge as the most influential variables with relatively high importance values. Attitudes toward AI (AT) demonstrates the highest total effect, indicating that students’ positive perceptions and acceptance of AI play a key role in enhancing creativity. At the same time, AT also shows relatively high performance, suggesting that students generally exhibit favorable attitudes toward AI, and further strengthening this dimension may generate additional creative benefits.

Digital competencies (DC) also shows a strong importance level, ranking second among all predictors. However, its performance is only moderate relative to AT, implying that while digital competence is a critical driver of creativity, there remains substantial room for improvement. Strengthening students’ digital skills may therefore produce meaningful gains in creative outcomes.

In contrast, AI used for learning (AL) displays moderate performance but lower importance, suggesting that while students frequently use AI for academic purposes, its direct impact on creativity is less substantial than attitudinal or skill-related factors. This indicates that general learning-related AI use may not automatically translate into higher creativity without supportive cognitive or motivational mechanisms.

Finally, AI used for entertainment (AE) exhibits both lower importance and lower performance, making it the least influential predictor of student creativity. This suggests that entertainment-oriented AI use contributes minimally to creativity enhancement and should not be prioritized in instructional design or student development strategies.

Taken together, the IPMA highlights AT and DC as priority development areas, with attitudes offering the strongest leverage and digital competencies representing a high-impact but underdeveloped dimension. Conversely, AL and AE provide limited value for improving student creativity from an importance–performance perspective.

### 4.6 Endogeneity

A potential concern in observational studies is endogeneity, as more creative students may also be more inclined to adopt AI tools, making reverse causality plausible. To address this concern, we applied the Gaussian copula approach (53) to test whether AI used for learning (AL) and AI used for entertainment (AE) are endogenous. Specifically, copula terms were generated for AL and AE by transforming them into normal scores based on their empirical distribution, and these terms were included in the regression models. The results showed that none of the copula terms were statistically significant (*p* > 0.10), suggesting no evidence of endogeneity in the current study. Nevertheless, given the cross-sectional nature of the data, reverse causality cannot be fully ruled out. Future research should employ longitudinal or experimental designs, or instrumental variables, to further mitigate endogeneity concerns.

### 4.7 Robustness checks

To further ensure the robustness of our findings, we conducted three additional analyses. First, we included all control variables (Model 1), and the main associations, indirect effects via digital competencies, and moderating effects remained consistent with the baseline model. Second, we selectively removed control variables that had a significant effect on creativity (Model 2), and third, we removed those that were not significant (Model 3). In both cases, the results were consistent with the original theoretical model. These analyses indicate that the findings are highly robust and not driven by the inclusion of specific moderating or control variables.

## 5 Discussion

Before interpreting the findings, it is important to clarify that this study is conducted within a specific educational context, namely undergraduate students majoring in Traditional Chinese Medicine (TCM) in one region of China. Therefore, the conclusions should be understood as context-dependent rather than universally generalizable to all higher education settings or AI-supported learning environments.

### 5.1 Key findings

The present study explores the relationship between AI use, digital competencies, and student creativity among TCM students in China. Our findings reveal several important insights that contribute to the existing literature on AI’s role in education and creativity development. Firstly, we found that AI used for learning (AL) and AI used for entertainment (AE) both positively associated with digital competencies (DC) among TCM students. Specifically, AL positively positively associated with DC (*β* = 0.349, *T* = 4.281), and AE also positively associated with DC (*β* = 0.349, *T* = 5.120). This result is consistent with previous research that suggests AI can enhance digital skills through various applications in both academic and recreational contexts (43, 54). However, our study extends this understanding by demonstrating that even in a specialized field like TCM, AI’s dual role in learning and entertainment can effectively promote digital competencies. This finding highlights the versatility of AI as a tool for skill development across diverse educational domains.

Secondly, digital competencies were found to be positively associated with student creativity (*β* = 0.685, *T* = 13.155) within the context of TCM undergraduate education. This result aligns with the broader literature on the importance of digital skills in fostering creativity (37, 55). However, our study innovatively demonstrates that in the context of TCM education, digital competencies play a crucial mediating role between AI use and creativity. This suggests that the development of digital competencies through AI use is not just a standalone outcome but also a pathway to enhancing creativity in specialized fields. This mediating effect highlights the importance of integrating digital literacy into the curriculum of traditional disciplines to unlock creative potential. Moreover, our study identified differential mediating effects of digital competencies on the relationship between AI use and student creativity. Digital competencies completely mediated the relationship between AL and student creativity, while partially mediating the relationship between AE and student creativity. This nuanced finding differs from previous studies that often assume a uniform mediating effect across different types of AI use (56). Our results suggest that the associations between AI use and creativity via digital competencies vary depending on whether AI is used for learning or entertainment. This highlights the need for a more nuanced understanding of AI’s role in education, recognizing that different AI applications may have distinct pathways to influencing student outcomes.

Finally, the study uncovered the moderating role of attitudes toward AI in the relationship between AI use and digital competencies within the context of TCM education. Specifically, positive attitudes toward AI enhanced the association between AE and digital competencies, while negative attitudes dampened the association between AL and digital competencies. This finding contrasts with some existing research that assumes a consistently positive influence of positive attitudes on AI adoption (5, 57, 58). Our results suggest that the relationship between AI use and digital competencies is more complex and contingent on the specific type of AI application and the user’s attitude. This highlights the importance of addressing students’ attitudes toward AI in educational interventions to maximize the benefits of AI tools.

### 5.2 Theoretical implications

This study makes several important theoretical contributions by extending Social Cognitive Theory (SCT) and enriching the literature on AI in education and student creativity. First, this study extends SCT into AI-supported educational contexts by conceptualizing AI use as a differentiated environmental stimulus rather than a uniform technological factor. While SCT traditionally treats the environment as a general external influence, our findings show that different forms of AI use (learning-oriented versus entertainment-oriented) function in fundamentally different ways. This distinction demonstrates that the “environment” in SCT should not be viewed as homogeneous in technology-rich contexts, but instead as a set of heterogeneous digital affordances with distinct cognitive and motivational implications. By doing so, this study refines SCT by introducing a more granular understanding of technological environments.

Second, this study identifies digital competencies as a core mediating mechanism that transforms AI use into creative outcomes. Prior SCT-based research often emphasizes self-efficacy, motivation, or behavioral engagement as central cognitive mediators (5, 59). Our findings complement and extend this tradition by showing that in AI-driven learning environments, digital competencies represent a fundamental cognitive capability that enables individuals to interpret, evaluate, and productively utilize AI outputs. The complete mediation between AI used for learning and student creativity and the partial mediation between AI used for entertainment and creativity indicate that creativity does not emerge directly from AI use itself, but from students’ ability to cognitively appropriate AI through digital skills. This insight advances SCT by specifying a technology-centered cognitive pathway that has been largely overlooked in prior research.

Third, this study advances SCT by revealing the dual moderating role of attitudes toward AI. Existing studies generally assume that positive attitudes uniformly strengthen technology use outcomes (2, 32). However, our findings demonstrate a more nuanced pattern: positive attitudes enhance the effect of entertainment-oriented AI use on digital competencies, while weakening the effect of learning-oriented AI use. This dual moderation suggests that attitudes toward AI do not simply amplify environmental effects, but reshape how different technological environments are cognitively processed. This challenges the linear assumption embedded in much of the SCT-based technology literature and highlights the conditional and context-sensitive nature of personal beliefs in AI-enabled learning.

### 5.3 Practical implications

To better align practical implications with the empirical findings, this section is organized into three subsections corresponding to educators, curriculum designers, and policymakers. Each implication is directly grounded in the key results of this study, including the mediating role of digital competencies, the dual moderating role of attitudes toward AI, and the IPMA results that identify digital competencies and attitudes as the most influential factors for enhancing student creativity.

#### 5.3.1 Implications for educators

The findings of this study offer context-specific practical insights for educators working in practice-oriented and professionally specialized educational settings, particularly in TCM programs. The results show that both AI used for learning and AI used for entertainment are positively associated with students’ digital competencies, which in turn foster creativity. Moreover, the IPMA results indicate that digital competencies and attitudes toward AI are the most important predictors of student creativity. Therefore, educators should prioritize instructional strategies that strengthen students’ digital skills while simultaneously shaping constructive attitudes toward AI.

First, educators are encouraged to integrate AI tools into both learning and auxiliary activities within the TCM undergraduate context. By demonstrating a positive association between AI use and digital competencies, this study provides empirical support for incorporating AI into teaching practices in specialized disciplines where traditional knowledge must be combined with digital technologies. For instance, TCM students can utilize AI-powered tongue and pulse diagnosis simulation platforms that analyze real-time images and simulate pulse sensations. By repeatedly practicing diagnosis techniques and comparing their assessments with AI-generated diagnostic reports, students can improve their understanding of TCM diagnostic methods while developing proficiency in using digital diagnostic tools. Second, the moderating role of attitudes toward AI suggests that students’ perceptions critically shape the effectiveness of AI-supported learning. In particular, negative or skeptical attitudes may weaken the positive influence of AI used for learning on digital competencies. Thus, educators should design targeted interventions for students who hold negative attitudes toward AI. For example, interactive workshops, guided reflections, and real-world demonstrations of AI applications can help students recognize AI as a supportive and complementary tool rather than a threat to traditional professional expertise. Third, in the context of TCM education, educators may organize AI-Assisted TCM Diagnosis Debate workshops. During these sessions, students analyze real medical cases using both traditional TCM diagnostic methods and AI-based systems (e.g., tongue and face analysis tools), and then debate the advantages and limitations of each approach. This practice not only helps students develop a more balanced and critical understanding of AI but also reinforces the idea that AI complements rather than replaces human judgment. Such activities directly respond to the study’s finding that attitudes toward AI regulate how effectively AI use translates into digital competencies and creative outcomes.

#### 5.3.2 Implications for curriculum designers

The significant mediating role of digital competencies indicates that creativity does not arise directly from AI use itself, but from students’ ability to interpret, evaluate, and productively utilize AI outputs. Therefore, curriculum designers should treat digital competency development as a core objective in AI-supported education, especially in specialized professional programs such as TCM.

First, digital literacy programs should be systematically integrated into the TCM curriculum as a complementary component of traditional professional training. This integration aligns with the empirical finding that digital competencies fully or partially mediate the relationship between AI use and student creativity. For example, introducing AI diagnosis simulation tools such as TCM AI Assistant into clinical training courses can simultaneously enhance professional knowledge and digital skills. TCM AI Assistant allows students to input symptoms, tongue and pulse conditions, and other diagnostic information, and then generates syndrome differentiation and treatment suggestions based on large-scale databases of classical cases and modern clinical studies. Through this process, students deepen their understanding of TCM principles while learning to operate advanced digital tools and interpret data-driven results. Second, the IPMA results show that digital competencies have high importance but only moderate performance, suggesting substantial room for improvement. This implies that curriculum designers should allocate more instructional time and learning resources to structured training in AI literacy, data interpretation, and human–AI collaboration skills, rather than focusing solely on increasing students’ frequency of AI use. Third, project-based learning activities can be designed in which students apply AI to analyze classical TCM cases, compare AI-generated results with traditional diagnostic reasoning, and reflect on the differences. Such tasks directly operationalize the mediating mechanism identified in this study and strengthen students’ ability to cognitively appropriate AI for creative problem solving.

#### 5.3.3 Implications for policymakers and institutional leaders

At the institutional level, the IPMA results identify attitudes toward AI and digital competencies as priority leverage points for enhancing student creativity. Policymakers and university administrators should therefore invest in infrastructures and policies that promote both digital skill development and healthy attitudinal orientations toward AI.

First, institutions may consider investing in AI-based resources and training programs for TCM and similar professional programs. These initiatives can help equip students with the digital competencies required for modern healthcare and professional practice, responding directly to the study’s finding that digital competencies are the central transmission mechanism between AI use and creativity. Second, teacher training programs should be strengthened to help instructors recognize and manage students’ diverse attitudes toward AI. Educators should be trained not only in technical AI applications but also in pedagogical strategies that address resistance, anxiety, or over-dependence on AI. This aligns with the empirical evidence that attitudes toward AI have a dual and asymmetric moderating role in shaping AI’s educational effectiveness. Third, policymakers should avoid promoting AI merely as an efficiency-enhancing technology. Instead, AI should be framed as a cognitive and creative partner that requires critical engagement and reflective use. Such a policy orientation corresponds closely to the study’s core findings that creativity emerges through digital competencies and is conditionally shaped by students’ attitudes toward AI. Finally, although the practical implications are grounded in the TCM undergraduate context, they may provide tentative guidance for other practice-oriented disciplines such as medicine, nursing, pharmacy, and engineering. However, these implications should be regarded as context-dependent, and their applicability to other educational systems or cultural settings requires further empirical validation.

### 5.4 Limitations and future research

This study provides insights into the association between AI and digital competencies and student creativity among TCM students, but it also has limitations. First, the generalizability of the findings should be interpreted with caution. Since the sample consists solely of undergraduate students majoring in Traditional Chinese Medicine at a single university in one region of China, the results cannot be directly generalized to other academic disciplines, educational levels, institutional types, or cultural contexts. Future studies should replicate this model across different majors, universities, and countries to examine its broader applicability. Second, the study relied on self-reported questionnaires, which may not fully capture the complexity of constructs like digital competencies and creativity. Incorporating qualitative interviews, performance-based assessments, or longitudinal observations could provide a more comprehensive understanding of these relationships. Third, a key limitation of this study is its cross-sectional design, which does not allow for causal inference. Accordingly, all findings should be interpreted as correlational rather than causal. Future research employing longitudinal or experimental designs is needed to establish causal relationships. Another limitation is that the study was not pre-registered, which may reduce the transparency and reproducibility of the research process. Future studies should adopt pre-registration practices, particularly when employing complex moderated-mediation models, to strengthen research rigor. Nevertheless, no *a priori* power analysis was conducted, which is a limitation. Future studies should incorporate pre-registration and a priori power analysis at the design stage to further strengthen methodological rigor. Additionally, the study focused on attitudes toward AI as a moderator but did not explore other potential moderating factors, such as individual differences, educational environment, or sociocultural influences (60). Future research should consider these factors to provide a more nuanced understanding of how AI impacts educational outcomes. Finally, AI may overthink simple problems into complex models, causing students to rely on technology rather than think independently (e.g., in TCM diagnosis, students directly apply AI recommendations and skip the core thinking training of syndrome differentiation and treatment). Future studies should address these aspects to better understand the ecological dynamics of AI integration in education. Future research should further explore how frontier technologies can be integrated to modernize TCM education. For example, future studies could investigate how generative AI may be leveraged to design intelligent diagnostic simulations that foster adaptive learning pathways and stimulate creative problem solving. Similarly, VR could be examined for its potential to build immersive anatomy or herbal medicine laboratories that enhance experiential and practice-oriented learning. In addition, blockchain warrants exploration for its role in securing the management of clinical training data and ensuring transparent credentialing of student competencies. Future research should integrate the “context–structure–mechanism–outcome” evolutionary paradigm of complex systems (61) to explore how emerging technologies (e.g., AI, VR, and blockchain) and human–AI collaboration contexts dynamically reshape cognitive structures and mechanisms, thereby driving nonlinear transformations in creativity and the modernization of TCM education.

## Data Availability

The raw data supporting the conclusions of this article will be made available by the authors, without undue reservation.
